# Impact of Risk Heterogeneity on the Feasibility of Hepatitis C Elimination Among People Who Inject Drugs: A Modelling Study

**DOI:** 10.1111/jvh.70096

**Published:** 2025-10-15

**Authors:** Kyra H. Grantz, Derek A. T. Cummings, Luis Mier‐y‐Teran Romero, Jacqueline Astemborski, Gregory D. Kirk, David L. Thomas, Javier A. Cepeda, Shruti H. Mehta, Amy Wesolowski

**Affiliations:** ^1^ Department of Epidemiology Johns Hopkins Bloomberg School of Public Health Baltimore Maryland USA; ^2^ Department of Biology and Emerging Pathogens Institute University of Florida Gainesville Florida USA; ^3^ Leidos Supporting the Administration for Strategic Preparedness and Response Washington DC USA; ^4^ Division of Infectious Diseases Johns Hopkins University School of Medicine Baltimore Maryland USA

**Keywords:** hepatitis C elimination, people who inject drugs, risk heterogeneity, transmission modelling, treatment‐as‐prevention

## Abstract

Although previous modelling work indicates treatment of < 10 people who inject drugs (PWID) per 100 person‐years (PY) could achieve hepatitis C virus (HCV) elimination targets in many settings, these models frequently make simplifying assumptions of heterogeneity in infection risk. Here, we evaluated the impact of incorporating risk heterogeneity in transmission models on the predicted effects of interventions and the feasibility of HCV elimination in high‐burden settings. We built an individual‐based model of HCV transmission informed by detailed data from a cohort of PWID in Baltimore, MD, including an individual‐ and time‐varying risk multiplier on the force of infection. We contrasted these risk‐informed models to risk‐agnostic models, ignoring this heterogeneity, and explored various levels of treatment and harm reduction scale‐up from 2020 to 2030. Risk‐agnostic models routinely estimated greater reductions in incidence (8%–19% higher for treatment rates of 10–90 per 100 PY) and greater numbers of infections averted per treatment course compared to otherwise equivalent populations modelled with risk heterogeneity. Elimination targets were only achieved in risk‐informed models when treating 90 PWID per 100 PY. Expanding harm reduction services dramatically improved the impact of elimination programs, particularly in averting new infections soon after treatment scale‐up initiation. Achieving HCV elimination targets among PWID in high‐burden settings will require substantial improvements in treatment access and harm reduction services. Models that ignore the unequal distribution of HCV risk, including the correlation between reinfection risk and onward transmission, can result in inappropriately optimistic estimates of the feasibility of elimination.

**Abbreviations**
ALIVE: AIDS linked to the intravenous experience
DAA: direct‐acting antiviral
HCV: hepatitis C virus
IQR: interquartile range
PWID: people who inject drugs
PY: person‐years

## 1 Introduction

Hepatitis C virus (HCV) infection results in high mortality and substantial health consequences, particularly among people who inject drugs (PWID) [1]. An estimated 6.1 million PWID are infected with HCV, and an estimated 43% of incident infections are attributed to the increased risk of transmission among PWID [2]. PWID are thus a critical population for the reduction or elimination of the HCV burden.

The introduction of highly effective, curative direct‐acting antiviral (DAA) treatments led to the establishment of elimination targets for HCV as a public health problem by 2030, including an 80% reduction in incidence and a 65% reduction in mortality from the 2015 baseline [3]. Increasing treatment uptake has been associated with reductions in HCV prevalence and HCV‐associated liver disease and mortality among PWID [4]. Achieving reductions in incidence, though, will require treatment scale‐up sufficient to provide indirect protection for untreated individuals through clearance of treated individuals and subsequent reductions in onward transmission [4, 5].

Despite the high acceptability and effectiveness of DAAs among PWID at the individual level [6], few studies have shown the population‐level indirect impacts of treatment scale‐up among PWID [6, 7]. Although prior studies have found evidence of reductions in HCV incidence in prison settings and in the incidence of HCV reinfection among people with HIV following treatment scale‐up, it is unclear how generalisable these findings are to a general PWID population, particularly in high‐burden settings [8, 9]. In the absence of empirical evidence, transmission modelling has been used to show that HCV elimination targets may be achieved among PWID in a variety of settings. Many studies found that treatment rates < 10 per 100 person‐years (PY) would be sufficient to achieve elimination, particularly when accompanied by the scale‐up of harm reduction programs [7]. However, other modelling studies indicate that higher treatment rates may be needed in certain settings [7, 10], and few places have achieved treatment scale‐up to the levels required to achieve elimination [11]. Even in Iceland and Georgia, where national elimination programs have greatly improved diagnosis and treatment, modelling indicates that further expansion will likely be needed to achieve elimination targets [11, 12, 13].

High rates of reinfection are a key challenge to effective elimination programs, and substantial reductions in incidence may hinge on the ability to both treat and continually prevent reinfection among key subpopulations of PWID that could sustain transmission chains in the entire population [14]. Although some models have shown that targeted treatment strategies to address those at high risk of reinfection are more effective than random treatment, most modelling work to date has made simplifying assumptions of either equal HCV acquisition and reinfection risk or coarse categorisations of risk (e.g., either engaged in active injection drug use or ceased) [7, 15]. In previous work in Baltimore, MD, we found that a small group of PWID remained at sustained higher risk of HCV acquisition for more than 10 years following entry into the study, including suggestive evidence that they have experienced higher reinfection rates following DAA treatment [16]. There is a need to better understand the feasibility of HCV elimination, particularly in light of unequally distributed and possibly increasing rates of reinfection as treatment uptake expands among higher risk populations [17].

Here, we sought to create an empirically grounded model of HCV transmission among PWID in Baltimore, Maryland, where HCV prevalence exceeds 60%. We first tested our hypothesis that incorporating individual and temporal heterogeneity in HCV risk modifies estimates of the population‐level impact of treatment compared to models that ignore risk heterogeneity. We then assessed the impact of various program designs on HCV prevalence and incidence, including different treatment levels, targeted treatment, short‐lived pulses of high treatment and scale‐up of harm reduction strategies to reduce reinfection. We sought to both evaluate common modelling structures and the level of complexity required in HCV transmission models and provide guidance for elimination program design in high‐burden settings.

## 2 Methods

**FIGURE 1:**
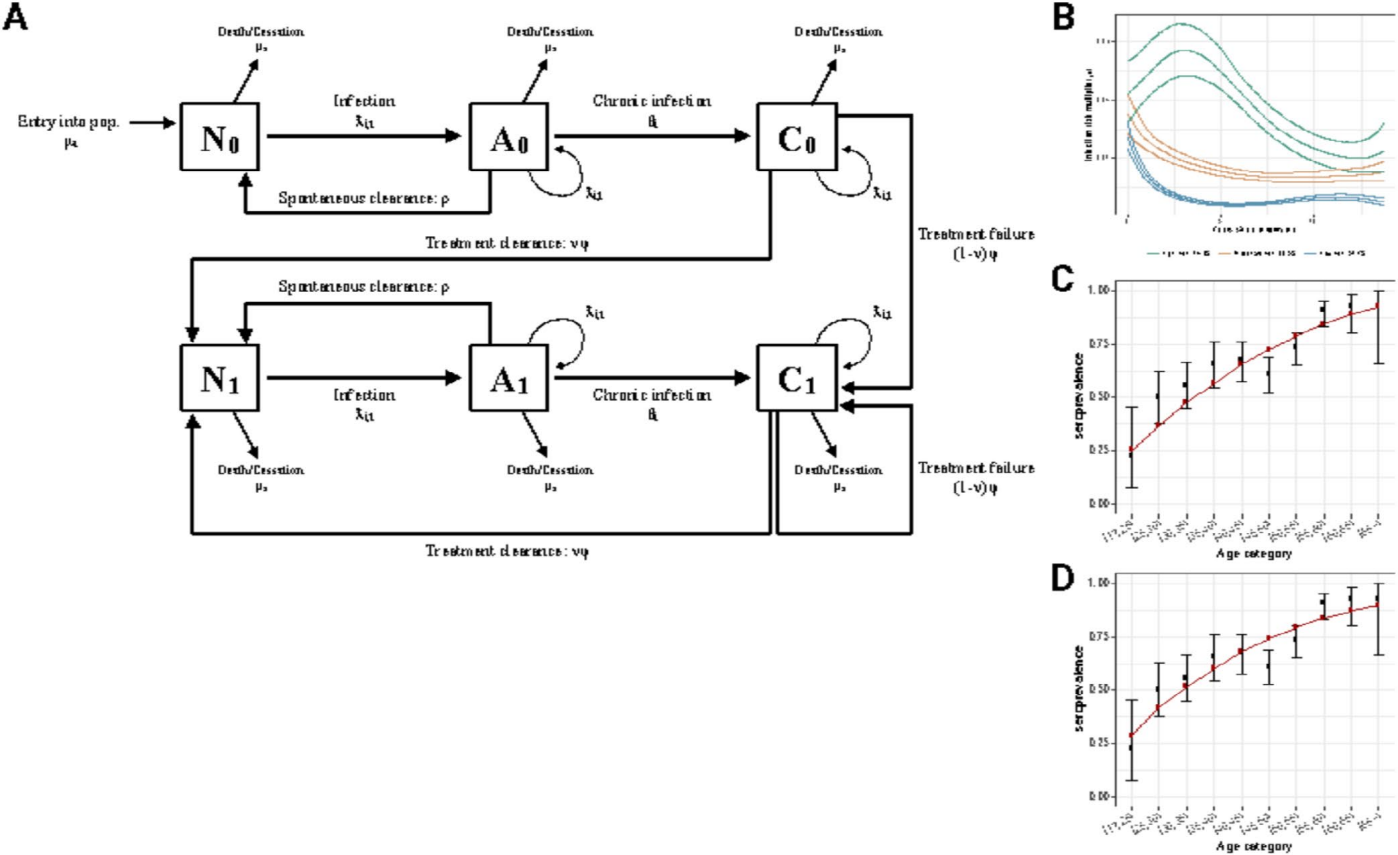
Overall modelling framework and parameterisation. (A) Model diagram of an individual‐based, discrete‐time, stochastic model of hepatitis C virus transmission among people who inject drugs. Individuals could belong to the HCV negative “N”, acutely infected “A”, or chronically infected “C” classes. Individuals with no prior infections, including those who newly initiate drug use, started in the N_0_ class. Individuals moved between states if they were infected or cleared the infection spontaneously or through direct‐acting antiviral treatment. Individuals enter the N_1_ class following successful treatment or the C_1_ class if treatment is unsuccessful, after which they are eligible for reinfection. Superinfections, or secondary infections acquired by individuals who are already infected, may occur while individuals are in the A or C classes. Parameters are defined in Appendix S1. (B) Risk class trajectories from which the individual‐ and time‐varying multiplier in risk‐informed models was derived. (C) and (D) Comparison of modelled seroprevalence (red lines) in 2015 compared to seroprevalence at baseline among individuals enrolled into the ALIVE cohort from 2015 to 2018 (black points, estimate; vertical lines, exact binomial confidence intervals) for the best‐fit transmission prefactors of (C) the risk‐agnostic and (D) the risk‐informed models.

We built an individual‐based, discrete‐time model of HCV transmission from 1990 to 2030 among PWID in Baltimore, MD, USA (Figure 1A; see Supporting Information). The model relied extensively on empirical characterisations of the AIDS Linked to the Intravenous Experience (ALIVE) study, a community‐based cohort of PWID recruited in successive enrollment periods from 1988 to 2018 (Figures S1–S7). Individuals in the model could be HCV negative, acutely infected, or chronically infected. Chronically infected individuals were eligible for DAA treatment beginning in 2015. We used age‐specific rates of long‐term injection drug use cessation [17, 18] and all‐cause mortality [19] to model turnover in the PWID population (average duration in population, 7.5 years) and age‐specific rates of shared injection partnerships [19, 20], along with current HCV prevalence, to determine the force of infection at each time point.

Critically, the model incorporated a multiplier on the force of infection, which could differ across individuals and time to represent the heterogeneous distribution of HCV acquisition risk in the population. On the basis of the prior analysis of ALIVE participants [16], the population in “risk‐informed” models was divided into three exclusive risk classes (high, comprised of 14.9% of the population; moderate, 33.5%; low, 51.7%), each with unique temporal trajectories representing the life‐course changes in risk behaviors from which the individual‐ and time‐varying multiplier values were drawn (Figure 1B, Table S1). For comparison, we ran scenarios where the risk multiplier was always equal to 1 to remove any individual or temporal heterogeneity (“risk‐agnostic” model). Given the use of different risk multipliers, we separately calibrated a time‐varying transmission prefactor for the risk‐informed and risk‐agnostic models to the same data on age‐specific seroprevalence (Figure 1C,D).

All models were run from 1990 to 2030 with biweekly time steps and a stochastic population of approximately 9500 PWID (Figure S1). The period 1990 to 2015 was used to calibrate age‐specific HCV seroprevalence (Figure 1C,D). The average prevalence of HCV viremia in the model in 2014, before any treatment was available, was 60.9%.

In all scenarios, the base treatment rate from 2015 to 2019 was 10 chronically infected PWID per 100 person‐years (PY), reflecting the rate of self‐reported treatment in this period among ALIVE participants. We then modelled multiple, constant rates of treatment from 2020 to 2030 (10, 30, 60, or 90 per 100 PY) to assess the potential impact of treatment‐as‐prevention. We modelled the effects of reducing the risk of reinfection among treated individuals (0%–50% reduction achieved, for example, through improved linkage to harm reduction services) [16, 21]. We also explored sensitivity analyses with risk‐assortative mixing (members of the same risk class 25% more likely to form an injection partnership) and with equal proportions of individuals in each risk class.

We estimated the reductions in prevalence and incidence of new infections (i.e., primary infections and reinfections of treatment‐cleared individuals) and all infections (including superinfections) from 2015 to 2030 and the number of incident HCV infections averted compared to scenarios with no treatment available. We presented the median estimate and interquartile range (IQR) from 250 stochastic simulations.

### 2.1 Ethics Statement

This analysis consisted of a secondary analysis of identifiable data collected in the ALIVE study. This work was approved under the ALIVE study by the Johns Hopkins Bloomberg School of Public Health Institutional Review Board (FWA #00000287).

## 3 Results

### 3.1 Incorporating Heterogeneity in HCV Risk Modified Effectiveness of Treatment Scale‐Up

Risk‐agnostic models that ignored heterogeneity in risk of HCV acquisition routinely estimated greater reductions in incidence from 2015 to 2030 and greater numbers of infections averted per treatment course delivered for otherwise equivalent populations modelled with risk heterogeneity. For example, at constant treatment of 10 per 100 PY, incidence in the risk‐agnostic model decreased 41.5% from 2015 to 2030 (IQR 34.3, 47.0), compared to 34.8% (IQR 28.7, 41.1) in the risk‐informed model, a relative difference of 19% (Table 1). Treatment of 60 per 100 PY achieved the incidence elimination target in the risk‐agnostic models (85.0% reduction from 2015 to 2030, IQR 82.8, 87.1), but treatment of 90 per 100 PY was required in the risk‐informed models. Inclusion of risk heterogeneity had less impact on the predicted reduction in prevalence (Table 1).

**TABLE 1:** Reductions from 2015 to 2030 in prevalence of active infection and in incidence of new infections, and total new infections averted per 1000 treatment courses delivered from 2015 to 2030 in scenarios with and without risk heterogeneity (risk‐informed and risk‐agnostic, respectively) and with different rates of direct‐acting antiviral treatment beginning in 2020. Negative infections averted indicate that more infections occurred in the treatment scenario than in the no‐treatment scenario. The total number of treatment courses delivered from 2015 to 2030 and the peak number of treatment courses delivered in any year are also presented.

Treatment	Model	Treatment courses delivered		Reductions, 2015–2030		New infections averted per 1000 treatment courses
Rate per 100 PY		Total, 2015–2030	Peak annual	Prevalence	Incidence of new infections	
		Median (IQR)	Median (IQR)	Median (IQR)	Median (IQR)	Median (IQR)
10	Risk‐agnostic	6073 (5976, 6176)	524 (509, 541)	37.8% (37.3, 38.5)	41.5% (34.3, 47.0)	3.2 (−10.7, 17.4)
	Risk‐informed	6155 (6064, 6276)	528 (514, 542)	37.1% (36.4, 38.0)	34.8% (28.7, 41.1)	−15.4 (−21.4, −9.4)
30	Risk‐agnostic	9366 (9226, 9524)	1115 (1088, 1142)	68.5% (67.9, 68.9)	70.9% (67.8, 74.7)	29.6 (20.5, 38.2)
	Risk‐informed	9531 (9388, 9694)	1124 (1095, 1153)	68.0% (67.4, 68.4)	63.0% (59.2, 67.0)	13.3 (9.1, 17.4)
60	Risk‐agnostic	11,052 (10,912, 11,263)	1952 (1918, 1999)	82.4% (82.0, 82.8)	85.0% (82.8, 87.1)	47.5 (39.9, 53.8)
	Risk‐informed	11,266 (11,091, 11,470)	1982 (1946, 2031)	81.9% (81.6, 82.3)	76.9% (73.9, 79.7)	33.8 (30.8, 37.0)
90	Risk‐agnostic	11,761 (11,606, 11,980)	2611 (2566, 2670)	87.7% (87.4, 88.0)	89.7% (88.1, 91.6)	55.0 (51.8, 58.1)
	Risk‐informed	11,965 (11,777, 12,173)	2649 (2606, 2706)	87.5% (87.1, 87.8)	82.9% (80.3, 85.2)	45.1 (41.8, 47.7)

**FIGURE 2:**
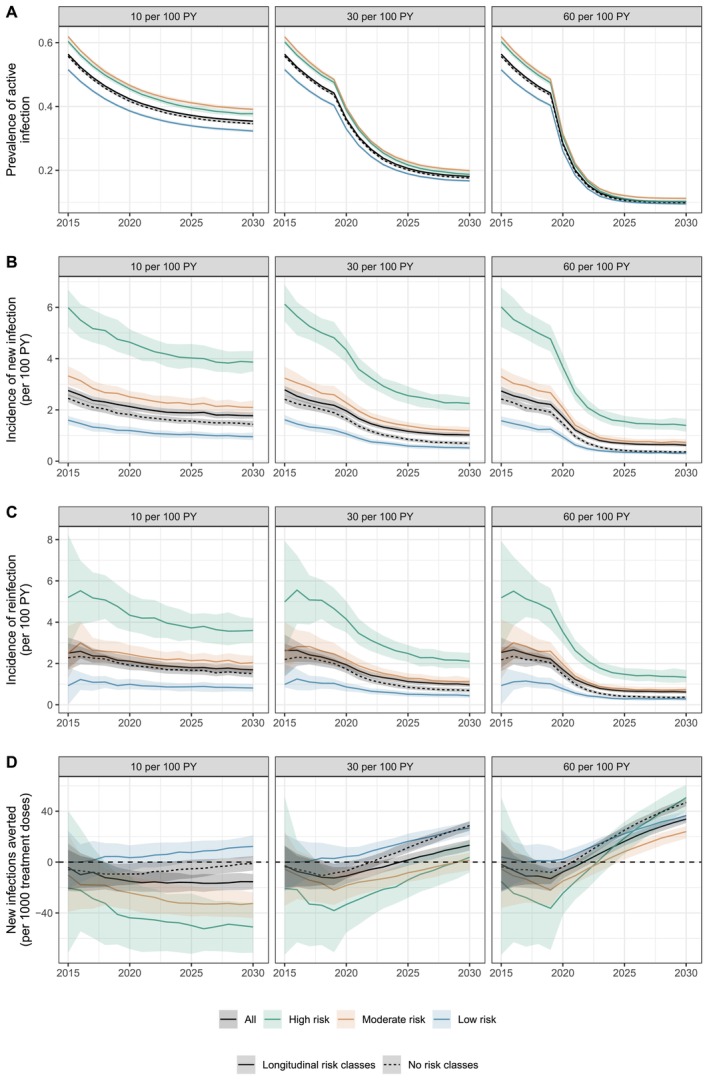
Simulation results with and without risk heterogeneity. (A) Prevalence of active infection, (B) incidence of new infections, (C) incidence of reinfection and (D) cumulative new infections averted per treatment courses in scenarios with and without risk heterogeneity. Lines represent median estimates, and shaded regions represent interquartile ranges across 250 simulations.

Incidence of all new infections and of reinfections was both routinely higher in the risk‐informed model relative to the risk‐agnostic model despite identical treatment rates (Figure 2, Figure S8). Although both scenarios had the same relative incidence of reinfection (compared to overall incidence), reinfection was concentrated among the high‐risk class in the risk‐informed model (Figures S9 and S10). The impact of this heterogeneous distribution of risk was particularly apparent at low treatment levels, where, on average, more new infections occurred from 2015 to 2030 with treatment of 10 per 100 PY than if there had been no treatment (Figure 2D). The limited reduction in prevalence at this treatment level, combined with the increasing number of PWID without active infection, led to higher and sustained rates of reinfection, particularly in the high‐ and moderate‐risk classes (Figure 2C, Tables S2 and S3). Sustained treatment rates of at least 30 per 100 PY were required for 5 years before treatment‐as‐prevention delivered a net positive impact on the occurrence of new infections (Figure 2D); this would require a median of 1124 (IQR 1095, 1153) treatment courses delivered in 2020, declining to a median of 560 (IQR 541, 579) annual courses in 2030 in a population of about 9500 PWID (Figure S8).

The number of infections (first, reinfection or superinfection) averted per treatment course was similar and positive across treatment scenarios (Table S2, Figure S11). We observed similar results, including differences between risk‐informed and risk‐agnostic models, in populations with a greater proportion in the highest risk class and with moderate levels of risk class‐assortative mixing (Figures S12 and S13).

### 3.2 Pulsed Treatment Strategies Could Be a More Efficient Use of Treatment Stocks

The required total and peak annual courses of treatment required to achieve the elimination target in risk‐informed scenarios may not be feasibly obtained or distributed in many contexts (Table 1). However, the relatively small increase in total treatment courses required when treating 90 versus 60 PWID per 100 PY illustrates that strategies delivering high rates of treatment over short periods of time may more efficiently reduce HCV burden by compounding early reductions in prevalence. We modelled multiple treatment pulses of 6 months, 1 year, or 2 years at rates of 60 or 80 per 100 PY beginning in 2020 in the risk‐informed model. Treatment was delivered at 10 per 100 PY outside of the pulse period from 2015 forward.

**FIGURE 3:**
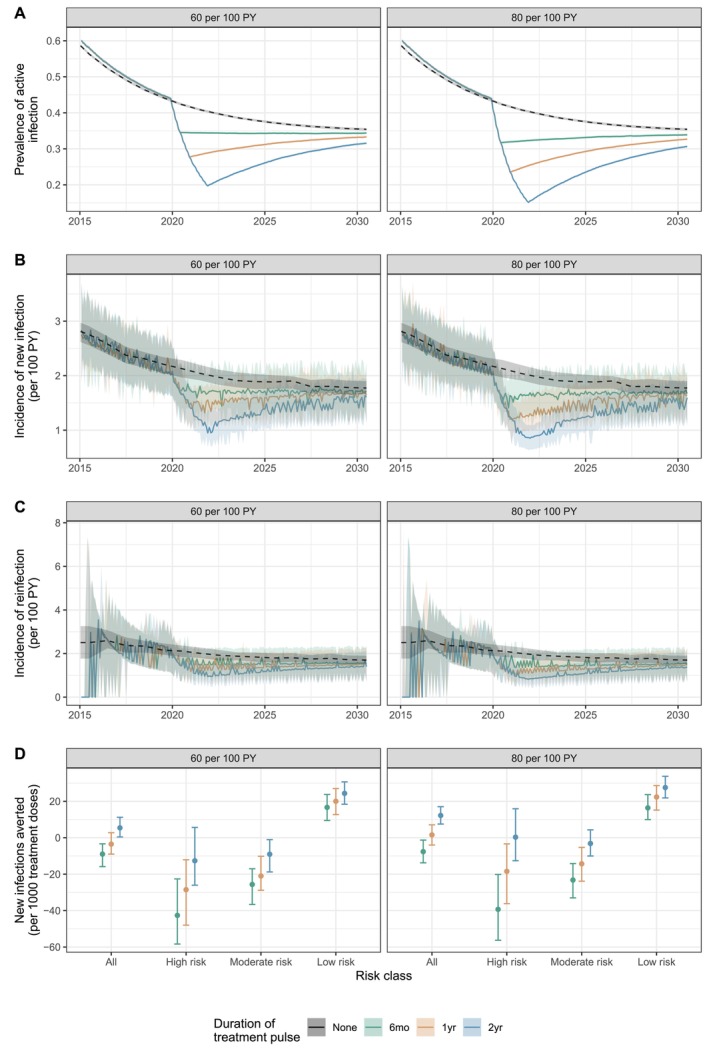
Simulation results using a treatment pulse strategy. (A) Prevalence of active infection, (B) incidence of new infections, (C) incidence of reinfection after treatment and (D) new infections averted from 2015 to 2030 per 1000 treatment courses delivered in scenarios with treatment pulses of either 60 or 80 courses per 100 PY for 6 months, 1 year, or 2 years, compared to a baseline scenario of constant treatment of 10 per 100 PY. Lines and points represent median estimates, and shaded regions and bars represent interquartile range across 250 simulations.

Though incidence and prevalence decreased sharply during the pulse period, they returned to nearly the same levels as a scenario with constant 10 per 100 PY treatment in less than 10 years after termination of the treatment pulse (Figure 3, Figure S26, Table S7). The transient reductions in prevalence and incidence, though, did lead to more infections averted, especially with longer pulses at higher treatment rates (Figure S25, Table S6). It was possible to avert a similar number of infections with a pulsed treatment strategy using fewer total treatment courses compared to scenarios with constant levels of treatment (Figure S27).

### 3.3 Targeting Treatment Had Limited Impact on Elimination

In scenarios where treatment was delivered equally to all risk classes, more infections were averted per course than in the low‐ or moderate‐risk class (Table S2). This pattern did not hold for new infections alone at lower treatment levels, where the increased clearance and susceptibility among high‐risk individuals led to more new infections.

Though logistically challenging to identify and target treatment to select subpopulations, the increased efficiency per course in the high‐risk population suggested targeting treatment could lead to greater population‐wide impacts. We modelled scenarios where the overall treatment rate remained constant, but high‐risk individuals were 33% or 66% more likely or 33% less likely (e.g., because of provider stigma or reduced engagement in care) to be treated than low‐ and moderate‐risk classes.

**FIGURE 4:**
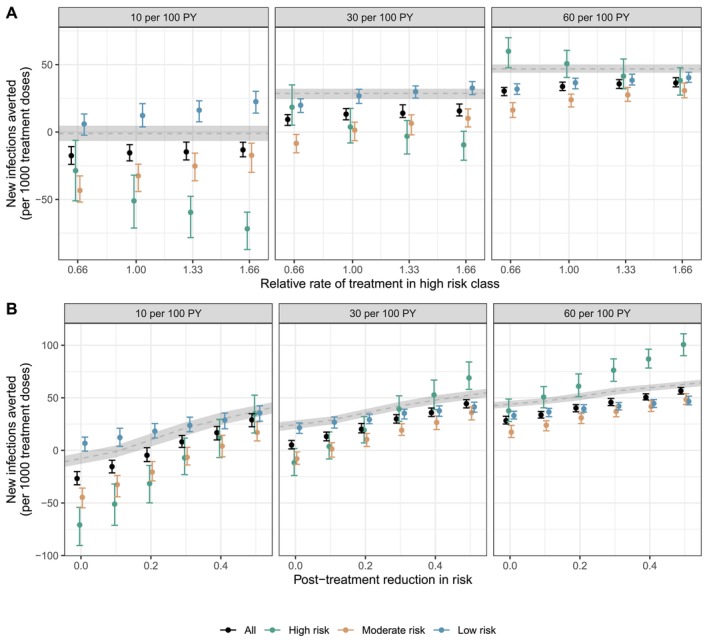
New infections averted per treatment course delivered, compared to scenarios with no treatment, by (A) the relative rate of treatment in the high‐risk class compared to the low‐ and moderate‐risk classes and (B) post‐treatment reductions in reinfection risk through harm reduction strategies. Points represent the median estimate, and vertical bars represent the interquartile range across 250 simulations. The dashed line and shaded region show the median and interquartile range for the model scenario without risk heterogeneity.

Targeted treatment in the high‐risk class slightly increased the overall efficiency of each course at preventing infection, especially at lower treatment levels (Figure 4A, Figures S14–S17). The greater reductions in prevalence in the high‐risk class when they were targeted for treatment paid dividends in reducing incident cases in the low‐ and moderate‐risk classes. However, the effect was modest and resulted in increased prevalence in the non‐targeted classes compared to the equal treatment scenarios (Figure S15).

The proportion of treated individuals who were reinfected increased as treatment was targeted towards higher risk individuals (Figure S10). Preferential clearance and susceptibility to reinfection among high‐risk individuals could give the appearance of a less successful program (i.e., more reinfection) at low or moderate treatment levels. The improvements in treatment efficiency with targeted treatment of high‐risk individuals were greater when there was class‐assortative mixing and when there was a larger high‐risk population (Figures S18–S22).

### 3.4 Preventing Reinfection Was Critical to Achieving Elimination Targets

Prior work has highlighted the need for combined treatment and harm reduction strategies to achieve noticeable reductions in HCV burden (7). Here, we focused solely on the impact of reducing the risk of reinfection post‐treatment, when PWID are already engaged in care and could be included in various wrap‐around services and harm reduction programs, rather than modeling population‐wide interventions.

Decreasing the risk of reinfection greatly improved the impact of treatment‐as‐prevention (Figure 4B, Figure S23). For example, with the treatment of 10 PWID per 100 PY, the reduction in incidence from 2015 to 2030 increased from 33.5% (IQR 26.2, 39.0) with no post‐treatment reduction in risk to 37.9% (IQR 32.5, 44.0) with a 20% reduction in reinfection risk, a 13.1% relative increase (Table S5). Most of the benefit of these additional interventions, especially at high treatment rates, occurred soon after treatment scale‐up began, when prevalence and thus reinfection rates were still high (Figure S24). Although the overall reductions in incidence were only modestly improved at high treatment levels (the reduction in incidence increased from 75.3% with no post‐treatment reductions to 78.0% with a 20% reduction in post‐treatment risk, a 3.5% relative increase, with the treatment of 60 per 100 PY), the early prevention of reinfection markedly increased the number of infections averted (Figure 4B, Table S4).

Substantially higher reductions in post‐treatment risk were needed in the risk‐informed models to achieve the same impacts as the risk‐agnostic models (Tables S4 and S5). Increasing the post‐treatment reductions in risk, and thus reducing the role of reinfection on transmission dynamics, also helped to close the gap between the predicted number of infections averted and reductions in the incidence of risk‐informed and risk‐agnostic models (Figure 4B).

## 4 Discussion

Achieving hepatitis C elimination targets among people who inject drugs in high‐burden settings will be difficult and require a multifaceted approach. In a model of PWID in Baltimore, Maryland, with a baseline prevalence of 60% that accounted for risk heterogeneity, we found that treatment of up to 90 infected PWID per 100 PY is needed to achieve the elimination target of 80% reduction in incidence by 2030. At current treatment levels around 10 per 100 PY, the large reservoir of HCV infection and accordingly high hazard of infection in this population results in only modest declines in incidence (35% from 2015 to 2030) and negligible indirect effects of treatment scale‐up; in fact, more new infections may occur than if no treatment had been delivered, as the susceptible pool of individuals is increased through treatment clearance. This scenario also required more than 6100 treatment courses, on average, in a population of 9500 PWID to be delivered by 2030. Though screening and treatment rates in the United States are improving, particularly following the end of Medicaid restrictions, many individual and structural barriers remain to increasing treatment uptake to the levels required to achieve elimination [22].

Elimination in a high‐burden setting will require treatment scale‐up along with reductions in the risk of reinfection through comprehensive harm reduction services. In our model, we observed that reducing reinfection risk specifically among PWID already engaged in care was highly effective at improving the indirect effects and overall impact of treatment scale‐up, independent of other reductions to the overall hazard of infection. The impact of reductions in reinfection risk was greatest when prevalence and thus reinfection rates are high; it will therefore be critical to couple any treatment scale‐up with simultaneous harm reduction services scale‐up to reduce reinfection.

A key strength of this work is the strong empirical foundation of the model, including information on individual heterogeneity and the temporal dynamics of HCV risk. We found that ignoring this skewed distribution of risk (i.e., assuming uniform reinfection risk among treated individuals) can result in inappropriately optimistic estimates of the impact of treatment scale‐up. That is, models that assume all individuals are equally likely to be reinfected fail to capture that the individuals most likely to be reinfected are also most likely to contribute to onward transmission (i.e., high‐risk individuals earlier in their injection careers and younger, when more injection partnerships are formed). This correlation is likely to attenuate the indirect protective effects of treatment scale‐up and thus result in smaller reductions in HCV incidence. That reducing the risk of reinfection made the risk‐informed and risk‐agnostic models appear more similar in their estimates of program impact, particularly of indirect effects like infections averted, is further evidence that the unequal distribution of reinfection can modify transmission dynamics and treatment impact.

Another key strength of this work is the estimation of indirect effects of treatment‐as‐prevention through infections averted. Evaluating only reductions in HCV incidence and prevalence by 2030 can mask intermediate benefits of targeted or pulsed treatment programs. We found that high levels of treatment delivered over a short period of time can lead to significant but transient reductions in prevalence and thus a greater number of infections averted versus delivering the same amount of treatment over a longer period. The ability to identify sufficient treatment‐eligible individuals and stockpile the number of treatment courses required for these pulses has not yet been demonstrated in the United States; however, it has required substantial investment and coordination in other countries [12, 13, 22]. These results suggest both the need to evaluate program impact beyond just reductions by 2030 and the potential for greater impact with more limited investment if a long‐acting treatment or vaccination could be quickly distributed at high coverage levels.

Our results differed noticeably from other modelling studies, which have typically found more sizable impacts at lower treatment rates, even with similar starting HCV prevalence [7, 23, 24, 25]. There are several possible explanations for these differences. First, individuals in this population spent an average of just 7.5 years in the population before long‐term cessation of injection drug use or death. Most models have longer average durations of injection drug use, and shorter durations (or equivalently, higher turnover in the PWID population) have been shown to decrease treatment impact [24, 25, 26]. Our model also incorporated larger and more granular differences between high, moderate and low‐risk individuals compared to previous models, which are categorised on the basis of high or low injection risk or coverage in harm reduction programs [7, 24, 25, 27]. We note, too, that our model estimates are broadly in line with the first empirical estimates of treatment‐as‐prevention effectiveness (the STOP‐C study), which observed a 48% reduction in incidence over 5 years following treatment of 70% of eligible participants in Australian prisons [8].

The estimates from this model were optimistic in that we assumed individuals were immediately eligible for treatment upon entering the chronic infection state. Increasing time to diagnosis and treatment can dramatically decrease the rate at which elimination targets are reached [8, 28]. Limited engagement in care and treatment uptake among certain individuals may also modify program impact. Although we found only modest impacts of limited or increased treatment among high‐risk individuals in our model, addressing the structural barriers and stigma in accessing treatment will be necessary to ensure broad, equitable impact of treatment programs [29]. We also lacked information here on the exact network of shared substance use partnerships in Baltimore. We used age‐specific injection partnerships as a proxy, though this implicitly assumed that all individuals have some probability of injecting with all other individuals in the population [20]. Previous work has found that network structure can be an important determinant of how effective treatment programs are and, in particular, whether network‐informed treatment strategies can be more effective than random treatment [15, 30]. Work exploring the individual network characteristics in the ALIVE cohort is ongoing and will be an important consideration in future modelling work.

Finally, our focus was on transmission‐related outcomes, rather than clinical outcomes including HCV‐associated morbidity and mortality, in a high transmission setting. Programs which prioritise reductions in HCV‐associated mortality would likely favour treating older, lower‐risk individuals with more advanced liver fibrosis or disease, which would in turn likely reduce the indirect effects of treatment on incidence [4]. Such programs would also likely favour constant rates of treatment targeted at individuals upon onset of more advanced disease, versus the high rates of constant or pulsed treatment which provided more efficient reductions in incidence in our model. In settings where indirect effects are likely to be negligible anyway, however, prioritising the clinical benefit of treatment may be preferred.

Although the estimates of program effectiveness provided here are specific to the modelled population of PWID in Baltimore, MD, our work has several important implications for future modelling work and other populations. Incorporating individual heterogeneity and temporal patterns in HCV risk reduced the modelled impact of treatment scale‐up compared to models that assumed uniform risk. This difference is due to concentrated reinfection events among high‐risk individuals when they are modelled separately. To the extent that data are available, it is critical to incorporate refined risk heterogeneity in models to fully capture the role of reinfection in treatment‐as‐prevention programs. Reducing reinfection following treatment, which offers a critical opportunity to engage PWID in integrated care, counselling, and harm reduction programs [4, 21], may be particularly effective at improving the impact of treatment‐as‐prevention and prove more feasible than population‐level harm reduction interventions. Although achieving HCV elimination targets will be challenging in high‐burden settings, treatment scale‐up combined with individual, provider, and system‐level interventions to improve uptake, adherence and prevent reinfection can have a substantial impact among HCV‐infected and uninfected PWID alike.

## Conflicts of Interest

The authors declare no conflicts of interest.

## Supporting information


**Data S1:** jvh70096‐sup‐0001‐DataS1.docx.

## Data Availability

The data that support the findings of this study are available on request from the corresponding author. The data are not publicly available due to privacy or ethical restrictions. Data Sharing: Model code is available at: https://doi.org/10.5281/zenodo.14602203. Proposals should be directed to kgrantz@jhu.edu and awesolowski@jhu.edu; requesters will need to sign a data access agreement.
