# Supplementary material for: Impact of Risk Heterogeneity on the Feasibility of Hepatitis C Elimination Among People Who Inject Drugs: A Modelling Study
Source: J Viral Hepat. 2025 Oct 15;32(11):e70096. doi: 10.1111/jvh.70096 (PMC12522077; doi:10.1111/jvh.70096)
Supplement: Supplementary file 1 — Data S1: jvh70096‐sup‐0001‐DataS1.docx. [file JVH-32-0-s001.docx]

**Appendix 1. Supplementary Information**

**METHODS**

*Model description*

*Transmission and disease progression.*

We built an individual-based, discrete-time, stochastic model of Hepatitis C virus (HCV) transmission among people who inject drugs. The model relies extensively on empirical characterizations of the ALIVE cohort, a study of PWID enrolled in successive enrollment periods from 1988 to 2015 in Baltimore, MD USA. All models were run for the time period 1990 ($t=0$) to 2030 with biweekly time steps, $\delta t$.

Each individual $i$ could belong to one of three states in this model: HCV RNA negative ($N$), HCV acute infection ($A$), and HCV chronic infection ($C$). Individuals who have never been treated for HCV infection are designated as belonging to $N_{0}$, $A_{0}$, or $C_{0}$. Those who have been treated for HCV infection belong to to $N_{1}$, $A_{1}$, or $C_{1}$. For notational purposes, we define $\epsilon_{i,t}=1$ if an individual has ever been treated before time $t$ and $=0$ otherwise. Each individual belongs to one of three risk classes, $c\in\{1, 2, 3\}$, based on a prior analysis of latent classes of HCV acquisition risk, from which a class- and time-varying risk class multiplier on the force of infection $\gamma_{c,i,t}$ is derived (Figure S4).

There are four key transmission and disease progression processes which can occur at each time step: (1) treatment, (2) progression to chronic infection, (3) spontaneous clearance; and (4) infection. Rarely, multiple events could occur at each time step for each individual; in this case, infection was assumed to be the last event to occur (e.g., treatment-associated or spontaneous clearance was assumed to occur first, followed by new infection).

*(1) Treatment.* Individuals in state $C$ are eligible for HCV treatment at risk class- and time-varying rate $\phi_{c,t}$. This rate is converted into a per-time step probability from which random binomial draws are conducted:

$$\Pr\left( \text{treatment} \right)=1-\exp\left( -\delta t*\phi_{c,t} \right)$$

Those who achieve sustained virologic response (SVR) with probability $\nu$ move into state $N$; those who do not clear infection remain in state $C$:

$$\Pr\left( \text{SVR} | \text{treatment} \right)= \nu$$

Following first treatment ($\epsilon_{i,t}=1$), there is an assumed reduction in transmission by $\alpha_{i}$.

*(2) Progression to chronic infection.* Each individual has a pre-specified duration of acute infection, $\theta_{i}$, after which they will progress to chronic infection in state $C$. The duration of infection is unaffected by any possible superinfections that may occur while an individual is in the acute infection state $A$; that is, the duration of acute infection begins upon first entry into $A$.

*(3) Spontaneous clearance.* Once in state $A$, individuals may spontaneously clear their infection. We model individual heterogeneity in the probability of clearance by assigning each individual a constant probability of clearance $\rho_{i}$ from a skewed distribution (see Parameterization and calibration, below). In this way, individuals who clear once are typically more likely to clear infection again.

As $\rho_{i}$ represents the overall probability that an individual will spontaneously clear their infection while in the acute infection stage, we consider the rate of clearance to be $\rho_{i}/\theta_{i}$. Hence, the probability of clearance at each time step is:

$$\text{Pr}(\text{spontaneous clearance}) = 1 - \text{exp}( -\delta t * \rho_{i}/\theta_{i})$$

*(4) Infection.* All individuals are at risk of infection. Individuals in states $A$ and $C$ are infectious. Individuals in state $N$ move to state $A$ once infected and become eligible for spontaneous clearance or progression to chronic infection. Individuals in states $A$ or $C$ remain in their same state following infection; that is, superinfections do not affect model state or duration in acute infection stage. Recall that individuals are assigned a time-varying risk multiplier, $\gamma_{c,i,t}$, and experience an assumed reduction in transmission once treated, $\alpha_{i}$.

A time- and risk class-invariant age-specific mixing matrix $K_{a_{i},a_{j}}$dictates the relative rate with which infectious contacts are made between individuals of age $a_{i}$ and $a_{j}$. We fit a time-varying transmission prefactor $\beta_{t}$ (see details below). Therefore, the force of infection $\lambda_{i,t}$ for individual $i$ at time $t$, where $I$ represents the set of infectious individuals in states $A$ and $C$ at time $t$, is:

$$\lambda_{i,t}=\left( 1-\epsilon_{i,t}\alpha_{i} \right)*\beta_{t}\gamma_{c,t,i}\sum_{j\in I} \left[ \left( 1-\epsilon_{j,t}\alpha_{j} \right)*\gamma_{c,t,j}K_{a_{i,t},a_{j,t}} \right]$$

The force of infection is used to generate the probability of infection at each time step of duration $\delta t$, from which random draws are taken to determine whether an individual is infected:

$$\Pr(\text{infection})=1-\exp\left( -\delta t*\lambda_{i,t} \right)$$

*Entering and leaving the population*

Each population began with 10,000 simulated individuals entering the population in 1990 (if prevalent case) or soon after (if new user; see below). Individuals leave the population due to long-term cessation of injection drug or death at age-specific rate $\mu_{a}$, independent of current or former HCV status. 250 populations were simulated, in which the population size remains roughly constant over time (Figure S1A).

Figure S1. (A) Modeled population size at each time step for 25 randomly selected simulations; (B) the distribution of duration in population (time from entry into population to long-term cessation of injection drug use or death) and (C) the age distribution of PWID present in the population at 1990, 2015, and 2030 for 25 randomly selected simulations (black lines) compared to the age distribution of ALIVE participants enrolled from 2015-2018 (red line).

The age-specific rate of long-term cessation, $\mu_{\text{cessation},a}$, is derived from a prior analysis of initiation and cessation patterns among ALIVE participants (Figure S2A)^1^. Specifically, three classes of individuals who enter long-term cessation use (i.e., cessation of all injection drug use for ≥5 years with no evidence of relapse) at varying rates were identified: early cessation (19% of population; average time to cessation, 5 years since enrollment), delayed cessation (16% of population, average 9 years since enrollment), and late cessation (18% of population, average 14 years since enrollment). The remaining population experienced either sustained injection or variable, short-term cessation without evidence of long-term cessation.

There is limited data on how cessation patterns change with age; we start by assuming that the probability of belonging to the early cessation group, $p_{\text{early},a}$ increases by 2% absolutely with each 5-year age category. We then assume that the odds of belonging to the early cessation group compared to the delayed cessation group are 1·033 times higher per age category, based on prior analysis. These assumptions maintain the marginal probability of belonging to the early cessation group (0.19) and delayed cessation group (0·16). We assume the probability of late cessation $p_{\text{late},a}$ is 0·18 for all age categories, again based on earlier findings that age was not associated with membership in the late cessation class.

We use these assumptions to calculate the age-specific cessation rate, given the above durations to cessation:

$$\mu_{\text{cessation},a}=\frac{1}{5}p_{\text{early},a}+\frac{1}{9}p_{\text{delayed},a}+\frac{1}{14}p_{\text{late},a}$$

The age-specific cessation rates are combined with empirical, age-specific all-cause mortality rates of PWID in the ALIVE cohort, $\mu_{\text{death},a}$, to generate total age-specific rates of leaving the population (Figure S2)^2^. As in prior analysis, we estimated the all-cause mortality (including overdoses) in three time periods: 1988-1997, 1998-2005, and 2006-2018^2^.

At each time-step, independent binomial events are drawn for each individual in the population at time $t$, $P_{t}$, to determine whether that individual leaves the population. Note that, while we do model long-term cessation as permanent removal from the population, we do not model short-term injection drug use initiation and cessation dynamics. Temporal changes in substance use patterns, and therefore risk of HCV infection, are absorbed into the time-varying risk term $\gamma_{c,i,t}$.

$$\mu_{a}= \mu_{\text{death}, a}+ \mu_{\text{cessation},a}$$

$$\Pr\left( \text{leaving population} \right)=p_{\mu,i,t}=1-\exp\left( -\delta t*\mu_{a} \right)$$

$$\text{\#}\text{ leaving population}=\sum_{i\in P_{t}} \text{Binom}_{i}\left( 1,p_{\mu,i,t} \right)$$

Individuals enter the population at the same rate as individuals leave the population, in expectation. At each time step, a second independent binomial event is drawn for each individual in the population to determine whether and how many individuals should be created at each time step. The population size therefore can fluctuate but should remain roughly constant over time.

$$\text{\#}\text{ entering population}=\sum_{j\in P_{t}} \text{Binom}_{j}\left( 1,p_{\mu,j,t} \right)$$


Figure S2. (A) Age-specific annual long-term injection drug use cessation rates, (B) all-cause annual mortality rates, and (C) combined annual cessation and all-cause mortality rates of people who inject drugs, derived from the ALIVE cohort.

Individuals are assigned an age at entry $a_{t,i}$ and age at first injection drug use $a_{\text{IDU},i}$, based on the risk class-specific empirical distributions of age at enrollment into the ALIVE cohort and self-reported age of first injection drug use, respectively (Figure fS3; see section “Parameterization and calibration” for further details on risk classes). Individuals whose age at entry into the population is less than their age at first injection drug use ($a_{t,i} \leq a_{\text{IDU},i}$) are considered to be "newly initiated". These individuals were not previously at risk of HCV infection and therefore enter into model state $N_{0}$ as HCV seronegative and uninfected. These individuals do not enter the population until they have initiated injection drug use at time $t^{*} s$uch that $a_{t^{*},i}=a_{\text{IDU},i}$. Time since entry into the population, $\tau_{i,t}$ is equal to $0$ at $t^{*}$.

Individuals whose age at entry is greater than their age at first injection drug use ($a_{t,i}>a_{\text{IDU},i}$) are considered "prevalent entries". These individuals can be considered to have initiated injection drug use prior to entry and migrated into this population at time $t$. The HCV serostatus of these individuals is randomly drawn from risk class- and age-specific empirical probabilities of being HCV positive at enrollment into the ALIVE cohort. To determine whether seropositive individuals have active HCV infection at entry, a second random binomial draw is conducted based on the empirical conditional probability of having detectable HCV viremia at enrollment into the ALIVE cohort, given a positive HCV antibody test. Individuals who are seropositive and have an active HCV infection enter into model state $C_{0}$; all other individuals enter into model state $N_{0}$. Time since entry into the population, $\tau_{i,t}$ is rqual to $0$ at $t$ for “prevalent entries”.

Thus, the average duration in the population (that is, time from entry into the population until long term cessation of injection drug use or death) is 7·5 years, with a long tail (Figure S1B). The age distribution of PWID in the modeled populations approximates the age distribution of participants enrolled into the ALIVE cohort from 2015 to 2018, although the modeled population is slightly older (Figure S1C).

Figure fS3. Empirical distributions of (A) age at first injection drug use (IDU) and (B) age at enrollment into the ALIVE cohort among individuals enrolled 2005-2008 or 2015-2018 included in a prior analysis of HCV risk classes.

*Parameterization and calibration.*

*HCV risk classes and temporal trends.*

The probability of belonging to a risk class $c$, $p_{c}$, is equal to the empirical proportion of individuals belonging to each class from a prior analysis of HCV acquisition risk among ALIVE participants (Table S1, Figure S4). Briefly, we fit a random forest model of the discrete-time hazard, or probability, of first HCV acquisition at each ALIVE study visit among participants who were seronegative at baseline; the predictors included sociodemographic characteristics, injection and non-injection substance use behaviors, and HIV and general health status. We then used the fitted random forest to predict the risk of any HCV infection at all study visits for all participants, regardless of HCV status at enrollment, and fit a latent class mixed model to estimate classes of longitudinal trends in HCV acquisition risk. The composite HCV risk score estimated by the random forest discriminated well between individuals who did and did not seroconvert, and individuals assigned to the high-risk latent class had higher rates of seroconversion and post-treatment HCV viremia. Thus, these classes appear to reflect temporal patterns in risk of primary HCV infection and reinfection well.

From this analysis, we found 14·9% of ALIVE participants belonged to a high-risk class (Class 1), 33·5% belonged to a moderate-risk class (Class 2), and 51·7% belonged to a low-risk class (Class 3). We also completed a sensitivity analysis where the population was simulated to have equal proportions (33%) in each risk class.

Figure S4. Risk trajectories $\gamma_{c,t}$ for each of three HCV risk classes estimated in a prior analysis. Lines show the mean latent class trajectory and shaded regions show the 95% confidence interval of the mean trajectory from which the distribution of each $\gamma_{c,t}$ is derived. Legend shows the proportion of population assigned to each class.

Each individual follows a risk trajectory indexed from time since entry into the population, $\tau_{i,t}$. At each time step, individuals have a randomly drawn risk multiplier $\gamma_{c,i,t}$, drawn from the class-specific mean latent class trajectory and its associated 95% confidence interval. (Note we use $t$ and $\tau$ somewhat interchangeably, as they are uniquely invertible: $\tau_{i,t}=t-t_{entry, i}$ for each person $i$.) Specifically, we assume that $\gamma_{c,\tau}$ follows a Normal distribution with mean equal to the empirical mean $\hat{\gamma_{c,\tau}}$ and standard deviation derived from the empirical confidence interval:

$$\gamma_{c,t}\sim\text{Norm}\left( \hat{\gamma_{c,\tau}},\frac{\hat{\gamma_{c,\tau}^{0.975}}-\hat{\gamma_{c,\tau}^{0.025}}}{Z_{0.95}} \right)$$

The risk trajectories were estimated for the first 13 years following enrollment (equivalent to entry into the study population). Hence, for all $\tau_{i,t}>13\text{y}$, we draw from the distribution of $\gamma_{c,13}$. We did not explicitly model turnover or movement between the risk classes here, which has been previously shown to modify program impact in models with simplified risk structure^3^. We note, however, that each risk class follows a longitudinal trajectory, and each individual follows their own trajectory samples from the class-specific trajectory, such that risk status is not static over time.

*Seropositivity and HCV status at entry*

The overall probability that a "prevalent entry" is seropositive is derived from the empirical, class-specific proportion of individuals who were HCV seropositive at enrollment (Table S1). The conditional probability of being viremic if seropositive at enrollment was similar across all risk classes; therefore, we use the average to define $\Pr\left( \text{viremia} | \text{seropositive} \right)$ = 0·758.

|  | **Risk class size, n**  **(column %)** | **Seroprevalence, n (%)** | **Viremic, n (%)** |
| --- | --- | --- | --- |
| **Class 1 (High risk)** | 125 (14·9) | 105/125 (84·0) | 29/39 (74·4) |
| **Class 2 (Moderate risk)** | 281 (33·5) | 244/281 (86·8) | 94/120 (78·3) |
| **Class 3 (Low risk)** | 434 (51·7) | 293/433 (67·7) | 115/155 (74·2) |

Table S1. Population size, HCV seroprevalence at enrollment, and proportion of seropositive individuals with HCV viremia (i.e., active infection) at enrollment by HCV risk class in the ALIVE cohort.

We estimated the age class-specific probability of seropositivity at entry by finding the parameter $b$ that minimizes the sum of squared errors when comparing the cumulative exponential function $1-\exp\left( -b*a \right)$ to the seroprevalence at baseline in each age class for individuals enrolled from 2005-2008 and from 2015-2018. We average the two parameters fit for each enrollment period, and then adjust the age-specific curve by its population-weighted mean and the seroprevalence in each risk to maintain the marginal risk class-specific probabilities (Figure S5).

Figure S5. Age-specific scalar on risk class-specific HCV seroprevalence at baseline (black line), as estimated from the scaled HCV seroprevalence data at enrollment in 2005-08 (green dots) and 2015-2018 (red dots). Vertical bars show 95% exact binomial confidence intervals of scaled seroprevalence within each age category.

*Spontaneous clearance and progression to chronic infection*

Each individual is assigned an overall probability of achieving spontaneous clearance, $\rho_{i}$. Previous reviews have identified that between 26-37% of individuals will spontaneously clear HCV infection shortly after exposure^4–6^. There is some evidence that clearance rates are lower in PWID, although there is concern of underestimation due to rapid reinfection if intervals between viremia measurements are long^4,5^.

Previous studies have found that individuals who previously cleared infection are less likely to have persistent viremia if infected again^7,8^. The mechanisms of this heterogeneity are unclear (e.g., whether due to some acquired or innate immune response), although there are individual genetic variants strongly associated with increased clearance^4^. We therefore draw $\rho_{i}$ from a skewed distribution, to model some individuals being consistently more likely to clear infection. We assume $\rho$follows a Beta distribution with average 0·36, with a 10th percentile of 0·1 and 90th percentile of 0·66 (Figure S6B).

$$\rho\sim\text{Beta}(1.5, 2.666)$$

Each individual is also assigned a unique duration of acute infection, $\theta_{i}$, during which spontaneous clearance may occur and after which the individual will progress to chronic infection. Based on previous studies and reviews of the natural history of HCV infection^9,10^, it was assumed that this duration is uniformly distributed between 60 and 240 days (2 to 8 months).

$$\theta\sim\text{Unif}(60, 240)$$


Figure S6. Distributions of (A) reductions in reinfection risk following treatment $\alpha$ and (B) probability of spontaneous clearance $\rho$.

*Treatment rates and success*

We vary the treatment rate, $\phi_{c,t}$, between model scenarios. In our prior analysis of longitudinal risk trajectories, we did not observe differences in treatment rates between risk classes; each class had self-reported treatment rates of roughly 10 per 100 person-years. We therefore use $\phi_{.,t}$ = 0·1 as the baseline treatment rate beginning in 2015 in scenarios where any treatment is available, and then increase or decrease treatment rates beginning in 2020 based on the scenarios we were exploring. In other simulations, we varied the class-specific rates of treatment to model targeted treatment or limited treatment access among high-risk individuals. Note that all individuals in the chronic treatment state $C$ are immediately eligible for treatment; this assumption is supported by multiple studies which have were high awareness, acceptability, and screening rates among PWID, particularly as substance use-associated insurance restrictions have been lifted^11–14^. Individuals in the acute infection stage $A$ were ineligible for treatment, following standard clinical guidelines. Due to the low levels of reported treatment in the ALIVE cohort prior to the DAA era, we did not include treatment prior to 2015 in this model.

Multiple studies have shown high rates of sustained virologic response in PWID, ranging from 87 to 91%^15,16^. A recent review and meta-analysis found no difference in SVR between recent (i.e., reporting injection use within last year) and non-recent PWID^17^. Therefore, we assumed a fixed probability of achieving SVR once treated of $\nu$=0·9 for individuals with monoinfections and superinfections alike.

*Post-treatment reductions in reinfection risk*

A recent review found mixed results for the effect of Hepatitis C treatment on subsequent injection practices^18^. While some studies found no changes in probability of injection drug use post-treatment compared to those who were not treated, other studies have found modest reductions in odds of any injection post-treatment (OR treated vs untreated, 0·89 for injection within 12 weeks of treatment, 0·95 for 2 years post-treatment) or even substantial reductions in injection among those who complete versus opted out of treatment (OR 0·18). The evidence for reductions in injection frequency is similarly unclear. Other studies have found modest reductions in syringe sharing behaviors after notification of HCV status, a signal that linkage to care and associated counseling and services can modify risk behaviors^19^, while still other studies found no effect of HCV status notification^20^.

It is therefore unclear to what extent, if any, receiving treatment modifies substance use behaviors and subsequent infection risk. We assume initially that receiving treatment reduces subsequent HCV transmission risk by 10%, on average for the remainder of that individual’s injection career. Each individual has a fixed post-treatment reduction $\alpha_{i}$ drawn from a Beta distribution with average 0·1, 10th percentile 0·011, and 90th percentile 0·226 (Figure S6A):

$$\alpha\sim\text{Beta}(1, 9)$$

We vary the average reduction in other model scenarios (0-50% average reduction) to model scale-up of harm reduction interventions to reduce risk of reinfection among treated individuals. Note that we do not explicitly model the impact of specific harm reduction measures (e.g., expanding syringe services programs), but instead assume that the modeled reductions would be achieved through some combination of harm reduction strategies known to reduce HCV risk.

*Age-specific mixing matrix*

We use data on injection partnerships collected through the STEP study in Baltimore, MD from 2005-2007 to estimate an age-specific mixing matrix. Specifically, we find the relative intensity with which different age groups interact, above or below expectations under the random mixing assumption. We do not incorporate information on injection or paraphernalia sharing frequency in the matrix, as those are incorporated into the risk multipliers $\gamma_{c,i,t}$, and instead focus only on the probabilities of partnership formation.

We first define $N_{a,i}$ as the STEP population size in age group $a_{i}$, $N_{a_{i}}^{*}$ as the ALIVE population size in age group $a_{i}$, and $c_{a_{i}, a_{j}}$ as the average number of reported injection partnerships between a respondent in age group $a_{i}$and contact in age group $a_{j}$collected in the STEP study. We scale the STEP per-capita number of reported partnerships by the ALIVE population to get an observed intensity of mixing between age groups $a_{i}$ and $a_{j}$ of the ALIVE population. We also average the ($a_{i}, a_{j})$ and ($a_{j}, a_{i})$ observations to make the matrix symmetrical:

$$O_{a_{i},a_{j}}=\frac{1}{2}\left( \frac{c_{a_{i}a_{j}}}{N_{a_{i}}}N_{a_{i}}^{*}+\frac{c_{a_{j}a_{i}}}{N_{a_{j}}}N_{a_{j}}^{*} \right)$$

We can then estimate the expected number of partnerships, $E_{a_{i},a_{j}}$, if mixing between age groups were random and each age group maintained its marginal per-capita number of partnerships $c_{a_{i}}$. We first estimate the probability of individuals in age group $a_{i}$ having any partnership, $p_{a_{i}}$. Under random mixing assumptions, the expected number of partnerships in ALIVE between age groups $a_{i}$ and $a_{j}$ would then be the product of the probability of forming any partnership for each age group and the overall number of partnerships:

$$p_{a,i}=\frac{c_{a_{i}}N_{a_{i}}^{*}}{\sum_{a_{k}} c_{a_{k}}N_{a_{k}}^{*}}$$

$$p_{a,j}=\frac{c_{a_{j}}N_{a_{j}}^{*}}{\sum_{a_{k}} c_{a_{k}}N_{a_{k}}^{*}}$$

$$E_{a_{i},a_{j}}=p_{a_{i}}p_{a_{j}}\sum_{a_{k}} c_{a_{k}}N_{a_{k}}^{*}=\frac{c_{a_{i}}c_{a_{j}}N_{a_{i}}^{*}N_{a_{j}}^{*}}{\sum_{a_{k}} c_{a_{k}}N_{a_{k}}^{*}}$$

We take the ratio of the observed and expected age-specific partnerships to generate the mixing matrix, $K_{a_{i},a_{j}}$, as the relative intensity or departures from random mixing:

$$K_{a_{i},a_{j}}=\frac{O_{a_{i},a_{j}}}{E_{a_{i},a_{j}}}$$

We estimated the matrix using 5-year age categories up to 55-60 years of age. Values for older categories were extrapolated from this oldest age category, retaining the same distance between categories (i.e., the 65-70 year age category mixes with 65-70 category at the same rate as 55-60 with 55-60 category). The final matrix shows strong age assortativity in partnership formation, particularly among the youngest and older individuals in the population (Figure S7).

**

Figure S7. Age-specific mixing matrix $K_{a_{i},a_{j}}$determining the relative intensity of forming injection partnerships (i.e., having potentially infectious contact) between individuals of different age groups.

*Fitting transmission prefactors.*

We calibrate the overall transmission in the model by fitting two values of $\beta_{t}$, at $t=0$ (start 1990) and $t=9135$ (end 2014). We assume that $\beta_{t}$ changes linearly as a function of time between $\beta_{0}$ and $\beta_{9135}$

and that $\beta_{t}=\beta_{9135}$ for all $t>9135$. We sought to maximize the likelihood function:

$$\mathcal{L}\left( \beta;X \right)=\prod_{i} \prod_{a} p_{a,i}^{x_{a}}\left( 1-p_{a,i} \right)^{N_{a}^{*}-x_{a}}$$

$$\mathcal{l}\left( \beta;X \right)=\log\mathcal{L}\left( \beta;\mathcal{X} \right)=\sum_{i} \sum_{a} x_{a}\log p_{a,i}+\left( N_{a}^{*}-x_{a} \right)\log\left( 1-p_{a,i} \right)$$

Where $\beta=\{\beta_{0},\beta_{9135}\}$, $p_{a,i}$ is the modeled seroprevalence in age group $a$ for iteration $i$ at $t=9135$, $N_{a}^{*}$ is the number of ALIVE participants in age group $a$ enrolled from 2015-2018 with a baseline HCV antibody measurement, and $x_{a}$ is the number within $N_{a}^{*}$ which had a positive antibody test. We performed an iterative grid search across many possible combinations of $\beta=\{\beta_{0},\beta_{9135}\}$, continually refined to explore the relevant parameter space, with 25 stochastic model iterations for each $\beta$ to find the set which maximized the likelihood function. We restricted to $\beta$ where $\beta_{0} > \beta_{9135}$, based on historic trends in ALIVE and in other studies in Baltimore which show declining prevalence and incidence.

We repeated the model fitting for scenarios with and without longitudinal trajectories, due to changes in $\gamma_{c,i,t}$ which influenced the force of infection and thus $p_{a,i}$. For the same reason, we also repeated the model fitting for scenarios where we introduced class-assortative mixing (i.e., an additional multiplier on $K_{a_{i},a_{j}}$ if $c_{i}=c_{j}$

*Scenarios and Outcomes*

*Model scenarios*

To test our hypothesis whether incorporation of risk heterogeneity modified transmission dynamics and the impact of treatment-as-prevention, we modeled multiple scenarios across the same sets of population first with the randomly drawn, individual- and time-varying risk multipliers $\gamma_{c,i,t}$ (“risk-informed”) and then with a fixed risk multiplier for all individuals and time points, $\gamma_{c,i,t}=\frac{\sum_{c,i,t} \gamma_{c,i,t}}{N}= \gamma^{*}$ (“risk-agnostic”).

We explored various treatment scale-up scenarios representing continuation of current treatment rates in the ALIVE cohort (10 per 100 person-years [PY]) up to 90 per 100 PY. In all scenarios, there was no treatment prior to 2015, and treatment from 2015 to 2019 of 10 per 100 PY, reflecting the rate of self-reported treatment among ALIVE participants. Treatment was then provided at a constant rate from 2020 to 2030 to all chronically infected individuals.

In baseline scenarios, the average reduction in reinfection risk was 10%. This was varied in 10% increments from 0% to 50% in other scenarios.

We also considered scenarios where the treatment rate in the high-risk class differed from the treatment rate in the low- and moderate-risk classes. The overall population treatment rate remained constant, but the rate in the high-risk class could be 0·66, 1·33, or 1·66 times the rate of the low- and moderate-risk classes.

Finally, we conducted sensitivity analyses where the rate of forming shared injection partnerships was 25% higher for individuals of the same risk class, and where there were equal proportions of the population distributed to each risk class.

*Model outcomes*

We calculated and compared several outcomes across model scenarios. All outcomes were estimated in the entire population and by each risk class. The demographic processes in this model (deaths, entries into the population, and individual heterogeneity in risk and disease progression) are independent of transmission and treatment processes. Hence, to control for the effects of demographic processes on transmission outcomes, each model scenario was run on the same set of 250 simulated populations. Different transmission and treatment scenarios were then directly compared to the counterfactual population under a different transmission or treatment scenario.

We calculated the reductions in prevalence of active infection (the proportion of PWID with viremia) and in incidence from 2015 to 2030 and compared to the WHO elimination target of 90% reduction in incidence. Here, we estimated both incidence of new infections (rate of infection among individuals in $N$) and incidence of any infection (rate of infection among all individuals, including superinfections of individuals in $A$ and $C$; note that this would be virtually impossible to observe or estimate in the real world).

The number of all infections and new infections averted per treatment course delivered was estimated by comparing each population directly to its counterpart scenario where no treatment was delivered. We also compared the number of infections averted per treatment course delivered as a measure of treatment efficiency and indirect protection.

We similarly estimated the relative incidence of all infections and new infections in each scenario compared to the counterfactual no-treatment scenario. Incidence of reinfection was calculated as both the rate of all infections among individuals who had been treated ($\epsilon_{i,t}=1$), including superinfections, and the rate of new infections among treated but uninfected individuals in $N$.

**TABLES AND FIGURES**

|  | **10 per 100 PY** | **30 per 100 PY** | **60 per 100 PY** | **90 per 100 PY** |
| --- | --- | --- | --- | --- |
| **New infections averted per 1000 courses** | |  |  |  |
| *Without risk heterogeneity:* | | | | |
| *All* | 3·2  (-10·7, 17·4) | 29·6  (20·5, 38·2) | 47·5  (39·9, 53·8) | 55·0  (51·8, 58·1) |
| *With risk heterogeneity:* | | | | |
| *All* | -15·4  (-21·4, -9·4) | 13·3  (9·1, 17·4) | 33·8  (30·8, 37·0) | 45·1  (41·8, 47·7) |
| *High risk* | -51·0  (-71·2, -32) | 3·8  (-8·1, 17·5) | 50·8  (40·5, 60·6) | 73·1  (62·6, 83·2) |
| *Moderate risk* | -32·5  (-44·1, -23·8) | 1·4  (-6·4, 7·3) | 24·0  (18·6, 28·2) | 36·1  (30·9, 41·2) |
| *Low risk* | 12·3  (3·8, 21·1) | 26·8  (21·2, 31·7) | 36·6  (32·1, 40·0) | 41·7  (38·1, 44·5) |
| **All infections averted per 1000 courses** | |  |  |  |
| *Without risk heterogeneity:* | | | | |
| *All* | 232·3  (220·9, 243·2) | 220·8  (213·5, 226·9) | 222·5  (217·2, 229·7) | 224·5  (219·4, 230·9) |
| *With risk heterogeneity:* | | | | |
| *All* | 229·0  (219·2, 242) | 225·8  (217·1, 233·3) | 234·5  (228·0, 241·3) | 240·5  (235·4, 246·5) |
| *High risk* | 468·5  (430·6, 502·6) | 462·9  (439·7, 489·7) | 488·7  (471·5, 510·8) | 505·5  (482·7, 525·3) |
| *Moderate risk* | 243·3  (225, 264·2) | 238·7  (227·5, 249·7) | 248·3  (239·4, 260·1) | 254·4  (245·3, 265·6) |
| *Low risk* | 130·6  (119·7, 143·6) | 129·2  (121·9, 136·5) | 133·4  (127·0, 139·3) | 136·6  (131·3, 141·0) |

Table S2. Total infections averted (new infections and all infections, including superinfections) from 2015 to 2030 per 1,000 treatment in model scenarios with and without risk heterogeneity. Estimates are median (interquartile range) across 250 simulations. Negative values indicate more infections occurred in the treatment scenario than in the no treatment scenario.

|  | **10 per 100 PY** | **30 per 100 PY** | **60 per 100 PY** | **90 per 100 PY** |
| --- | --- | --- | --- | --- |
| **Reductions in prevalence:** | | | | |
| *Without risk heterogeneity:* | | | | |
| *All* | 37·8%  (37·3, 38·5) | 68·5%  (67·9, 68·9) | 82·4%  (82·0, 82·8) | 87·7%  (87·4, 88·0) |
| *With risk heterogeneity:* | | | | |
| *All* | 37·1%  (36·4, 38·0) | 68·0%  (67·4, 68·4) | 81·9%  (81·6, 82·3) | 87·5%  (87·1, 87·8) |
| *High risk* | 37·7%  (36·0, 39·3) | 68·9%  (67·6, 70·1) | 82·9%  (82·0, 84·0) | 88·4%  (87·6, 89·1) |
| *Moderate risk* | 36·9%  (35·7, 38·0) | 67·8%  (66·9, 68·6) | 81·9%  (81·3, 82·6) | 87·4%  (86·9, 87·9) |
| *Low risk* | 37·2%  (36·3, 38·3) | 67·6%  (66·9, 68·5) | 81·6%  (81·1, 82·1) | 87·1%  (86·7, 87·5) |
| **Reductions in incidence of all infections:** | | | | |
| *Without risk heterogeneity:* | | | | |
| *All* | 46·3%  (42·1, 50·3) | 74·2%  (71·7, 76·5) | 86·6%  (84·9, 88·4) | 91·0%  (89·6, 92·3) |
| *With risk heterogeneity:* | | | | |
| *All* | 37·9%  (33·3, 41·8) | 64·8%  (62·1, 67·5) | 78·8%  (76·7, 81·0) | 84·3%  (82·8, 86·5) |
| *High risk* | 35·9%  (29·6, 41·8) | 63·3%  (59·2, 69·2) | 78·6%  (75·3, 82·5) | 83·6%  (80·8, 87·2) |
| *Moderate risk* | 38·2%  (31·4, 45·1) | 65·7%  (61·6, 69·3) | 78·5%  (75·0, 81·6) | 85·1%  (81·9, 87·4) |
| *Low risk* | 38·9%  (30·3, 45·8) | 64·2%  (59·1, 71·0) | 78·8%  (76·0, 83·0) | 84·8%  (81·1, 87·6) |
| **Reductions in incidence of new infections:** | | | | |
| *Without risk heterogeneity:* | | | | |
| *All* | 41·5%  (34·3, 47·0) | 70·9%  (67·8, 74·7) | 85·0%  (82·8, 87·1) | 89·7%  (88·1, 91·6) |
| *With risk heterogeneity:* | | | | |
| *All* | 34·8%  (28·7, 41·1) | 63·0%  (59·2, 67·0) | 76·9%  (73·9, 79·7) | 82·9%  (80·3, 85·2) |
| *High risk* | 36·6%  (26·3, 44·1) | 63·8%  (56·6, 69·5) | 76·8%  (71·6, 81·6) | 82·6%  (79·1, 86·6) |
| *Moderate risk* | 36·5%  (25·6, 46·4) | 63·9%  (57·6, 69·9) | 76·9%  (73·4, 81·7) | 84·1%  (79·7, 86·8) |
| *Low risk* | 39·8%  (26·3, 50·5) | 66·7%  (61·5, 73·2) | 80·5%  (75·8, 84·3) | 85·4%  (82·0, 88·5) |

Table S3. Reductions from 2015 to 2030 in prevalence, incidence of all infections (including superinfections), and incidence of new infections in model scenarios with and without risk heterogeneity. Estimates are median (interquartile range) across 250 simulations.

|  | **10 per 100 PY** | **30 per 100 PY** | **60 per 100 PY** |
| --- | --- | --- | --- |
| **New infections averted per 1000 courses** | | | |
| *Without risk heterogeneity:* | | | |
| *0·1* | 3·2 (-10·7, 17·4) | 29·6 (20·5, 38·2) | 47·5 (39·9, 53·8) |
| *With risk heterogeneity:* | | | |
| *0* | -26·7 (-32·7, -20·2) | 5·2 (1·4, 9·4) | 28·3 (25·1, 32·4) |
| *0·1* | -15·4 (-21·4, -9·4) | 13·3 (9·1, 17·4) | 33·8 (30·8, 37·0) |
| *0·2* | -4·6 (-10·6, 2·6) | 20·1 (16·7, 25·6) | 40·3 (36·7, 43·5) |
| *0·3* | 8·1 (2·8, 14·2) | 29·9 (25·9, 34·0) | 45·7 (42·6, 49·4) |
| *0·4* | 16·9 (10·0, 22·7) | 35·9 (32·2, 40·3) | 50·6 (48·0, 53·8) |
| *0·5* | 29·0 (22·5, 34·8) | 44·5 (40·4, 48·2) | 56·7 (53·6, 59·8) |
| **All infections averted per 1000 courses** | | | |
| *Without risk heterogeneity:* | | | |
| *0·1* | 232·3 (220·9, 243·2) | 220·8 (213·5, 226·9) | 222·5 (217·2, 229·7) |
| *With risk heterogeneity:* | | | |
| *0* | 214·8 (202·9, 226·2) | 215·0 (208·0, 222·7) | 227·6 (221·6, 234·6) |
| *0·1* | 229·0 (219·2, 242·0) | 225·8 (217·1, 233·3) | 234·5 (228·0, 241·3) |
| *0·2* | 244·7 (233·3, 254·6) | 236·1 (228·2, 243·0) | 242·5 (235·7, 249·4) |
| *0·3* | 262·2 (252·6, 273·5) | 247·4 (240·1, 255·6) | 249·4 (242·5, 256·4) |
| *0·4* | 273·4 (259·3, 285·4) | 255·3 (248·2, 262·9) | 255·4 (249·1, 262·7) |
| *0·5* | 289·9 (278·8, 301·2) | 266·1 (257·4, 273·5) | 263·6 (256·3, 270·0) |

Table S4. Total infections averted (new infections and all infections, including superinfections) from 2015 to 2030 per 1,000 treatment by the post-treatment reduction in HCV risk in scenarios with and without risk heterogeneity. Estimates are median (interquartile range) across 250 simulations.

|  | **10 per 100 PY** | **30 per 100 PY** | **60 per 100 PY** |
| --- | --- | --- | --- |
| **Reductions in prevalence:** | | | |
| *Without risk heterogeneity:* | | | |
| *0·1* | 37·8% (37·3, 38·5) | 68·5% (67·9, 68·9) | 82·4% (82·0, 82·8) |
| *With risk heterogeneity:* | | | |
| *0* | 36·9% (36·2, 37·6) | 67·7% (67·2, 68·2) | 81·9% (81·5, 82·3) |
| *0·1* | 37·1% (36·4, 38·0) | 68·0% (67·4, 68·4) | 81·9% (81·6, 82·3) |
| *0·2* | 37·5% (36·8, 38·2) | 68·1% (67·6, 68·5) | 82·2% (81·7, 82·5) |
| *0·3* | 38·0% (37·3, 38·6) | 68·2% (67·8, 68·7) | 82·3% (81·8, 82·6) |
| *0·4* | 38·2% (37·6, 39·0) | 68·5% (68·0, 69·0) | 82·3% (81·8, 82·7) |
| *0·5* | 38·5% (37·7, 39·2) | 68·6% (68·1, 69·1) | 82·4% (82·0, 82·8) |
| **Reductions in incidence of all infections:** | | | |
| *Without risk heterogeneity:* | | | |
| *0·1* | 46·3% (42·1, 50·3) | 74·2% (71·7, 76·5) | 86·6% (84·9, 88·4) |
| *With risk heterogeneity:* | | | |
| *0* | 35·5% (30·4, 39·8) | 62·8% (59·9, 65·9) | 77·5% (74·4, 79·4) |
| *0·1* | 37·9% (33·3, 41·8) | 64·8% (62·1, 67·5) | 78·8% (76·7, 81·0) |
| *0·2* | 39·8% (36·5, 43·5) | 66·8% (64·0, 70·2) | 80·0% (77·6, 82·1) |
| *0·3* | 42·6% (38·4, 46·2) | 68·5% (65·7, 71·5) | 81·8% (79·6, 83·7) |
| *0·4* | 44·0% (40·2, 47·1) | 70·4% (68·4, 73·9) | 82·6% (80·2, 84·3) |
| *0·5* | 45·4% (41·1, 49·9) | 72·2% (69·5, 74·4) | 84·2% (82·3, 85·8) |
| **Reductions in incidence of new infections:** | | | |
| *Without risk heterogeneity:* | | | |
| *0·1* | 41·5% (34·3, 47) | 70·9% (67·8, 74·7) | 85·0% (82·8, 87·1) |
| *With risk heterogeneity:* | | | |
| *0* | 33·5% (26·2, 39·0) | 60·8% (56·4, 64·8) | 75·3% (72·1, 77·7) |
| *0·1* | 34·8% (28·7, 41·1) | 63·0% (59·2, 67·0) | 76·9% (73·9, 79·7) |
| *0·2* | 37·9% (32·5, 44·0) | 64·4% (60·2, 68·5) | 78·0% (75·2, 80·6) |
| *0·3* | 41·8% (36·1, 46·4) | 67·0% (63·3, 70·5) | 80·5% (77·4, 82·7) |
| *0·4* | 43·9% (38·4, 48·4) | 69·6% (65·9, 72·8) | 81·3% (78·4, 83·6) |
| *0·5* | 47·0% (41·4, 51·8) | 71·4% (68·3, 74·3) | 83·0% (81·1, 84·7) |

Table S5. Reductions from 2015 to 2030 in prevalence, incidence of all infections (including superinfections), and incidence of new infections by the post-treatment reduction in HCV risk in scenarios with and without risk heterogeneity. Estimates are median (interquartile range) across 250 simulations.

| **Treatment pulse** | | **Treatment courses** | **All infections averted** | | **New infections averted** | |
| --- | --- | --- | --- | --- | --- | --- |
| *Rate (per 100 PY)* | *Duration* |  | *Total* | *per 1000 treatment courses* | *Total* | *per 1000 treatment courses* |
| No pulse | | 6,155  (6,065, 6,276) | 1,406  (1,338, 1,497) | 229·0 (219·2, 242·0) | -96  (-134, -57) | -15·4 (-21·4, -9·4) |
| 60 | 6mo | 6,724  (6,605, 6,842) | 1,617  (1,527, 1,685) | 239·9 (230·3, 249·7) | -60  (-106, -22) | -8·9 (-15·9, -3·3) |
|  | 1yr | 7,157  (7,056, 7,289) | 1,762  (1,673, 1,835) | 245·5 (235·7, 255·5) | -25  (-64, 20) | -3·5 (-9·0, 2·7) |
|  | 2yr | 7,777  (7,666, 7,942) | 1,972  (1,880, 2,036) | 252·9 (242·4, 261·7) | 43  (3, 90) | 5·4 (0·4, 11·2) |
| 80 | 6mo | 6,899  (6,813, 7,039) | 1,679  (1,579, 1,757) | 241·8 (230·2, 252·8) | -54  (-97, -9) | -7·6 (-13·8, -1·3) |
|  | 1yr | 7,443  (7,324, 7,583) | 1,856  (1,784, 1,939) | 250·0 (241·8, 257·5) | 12  (-30, 54) | 1·6 (-4·0, 7·1) |
|  | 2yr | 8,155  (8,012, 8,311) | 2,094  (2,003, 2,180) | 256·6 (247·1, 266·0) | 101  (62, 139) | 12·3 (7·5, 17·0) |
| 160 | 6mo | 7,496  (7,397, 7,669) | 1,881  (1,791, 1,966) | 250·4 (240·8, 260·4) | 15  (-24, 54) | 2·0 (-3·2, 7·4) |
|  | 1yr | 8,198  (8,078, 8,369) | 2,118  (2,026, 2,207) | 258·9 (247·8, 266·7) | 108  (67, 146) | 13·0 (8·2, 17·9) |
| 320 | 6mo | 8,205  (8,081, 8,374) | 2,129  (2,043, 2,220) | 259·7 (250·3, 268·2) | 121  (81, 158) | 14·6 (9·8, 19·2) |

Table S6. Total infections averted (new infections and all infections, including superinfections) from 2015 to 2030 per 1,000 treatment in scenarios with brief pulses (6 months, 1 year, or 2 years) of high treatment rates (60 or 80 per 100 PY) and low treatment (10 per 100 PY) otherwise. Estimates are median (interquartile range) across 250 simulations.

| **Treatment pulse** | | **Reductions, 2015-2030** | | |
| --- | --- | --- | --- | --- |
| *Rate* | *Duration* | *Prevalence* | *All infections* | *New infections* |
| No pulse |  | 37·1% (36·4, 38·0) | 37·9% (33·3, 41·8) | 34·8% (28·7, 41·1) |
| 60 per 100 PY | 6mo | 40·9% (40·3, 41·5) | 29·0% (19·0, 38·3) | 27·2% (11·7, 38·9) |
|  | 1yr | 42·9% (42·0, 43·5) | 30·3% (20·4, 39·3) | 28·8% (8·9, 40·0) |
|  | 2yr | 45·8% (45·2, 46·5) | 33·2% (23·9, 41·2) | 31·5% (18·8, 41·9) |
| 80 per 100 PY | 6mo | 41·8% (41·1, 42·4) | 29·1% (21·6, 38·3) | 27·3% (14·2, 39·7) |
|  | 1yr | 43·9% (43·2, 44·5) | 32·6% (23·6, 42·7) | 28·2% (17·4, 41·7) |
|  | 2yr | 47·5% (46·6, 48·0) | 36·5% (27·4, 44·6) | 31·5% (16·2, 44·6) |
| 160 per 100 PY | 6mo | 43·9% (43·3, 44·5) | 31·5% (21·6, 40·9) | 26·1% (12·5, 39·6) |
|  | 1yr | 46·8% (46·1, 47·4) | 35·9% (24·2, 42·3) | 30·5% (18·0, 43·6) |
| 320 per 100 PY | 6mo | 46·5% (45·8, 47·3) | 34·0% (25·0, 41·9) | 30·7% (17·0, 44·0) |

Table S7. Reductions from 2015 to 2030 in prevalence, incidence of all infections (including superinfections), and incidence of new infections in scenarios with brief pulses (6 months, 1 year, or 2 years) of high treatment rates (60 or 80 per 100 PY) and low treatment (10 per 100 PY) otherwise. Estimates are median (interquartile range) across 250 simulations.

Figure S8. (A) Incidence of all infections, (B) annual number of treatment courses delivered, and (C) annual rate of treatment per 100 PY in model scenarios with and without risk heterogeneity. Lines represent median estimates and shaded regions represent interquartile ranges across 250 simulations.

Figure S9. (A) Relative annual incidence of reinfection compared to any new infection and (B) the proportion of new infections which were reinfection each year in models scenarios with and without risk heterogeneity. Lines represent median estimates and shaded regions represent interquartile ranges across 250 simulations.

Figure S10. Proportion of all treated individuals who experience a new reinfection event by relative rate of treatment in the high-risk class compared to low- and moderate-risk classes. Points represent median estimate and vertical bars represent the interquartile range across 250 simulations. Dashed line and shaded region show the median and interquartile range of reductions for the model without risk heterogeneity.

Figure S11. Cumulative all infections averted (including superinfections) per 1,000 treatment courses delivered in scenarios with and without risk heterogeneity. Lines represent median estimate and shaded regions represent interquartile range across 250 simulations.

Figure S12. (A) Cumulative all infections (including superinfections) and (B) new infection averted per 1,000 treatment courses delivered in scenarios with and without risk heterogeneity with a greater proportion of population (33%) in the high-risk class. Lines represent median estimate and shaded regions represent interquartile range across 250 simulations.

Figure S13. (A) Cumulative all infections (including superinfections) and (B) new infection averted per 1,000 treatment courses delivered in scenarios with risk class-assortative mixing (within class contacts 25% more likely than between class contacts). Lines represent median estimate and shaded regions represent the interquartile range across 250 simulations.

Figure S14. All infections (including superinfections) averted per treatment courses delivered, compared to scenarios with no treatment, by (A) the relative rate of treatment in the high-risk class compared to the low- and moderate-risk classes and (B) post-treatment reductions in HCV risk through harm reduction strategies. Points represent median estimate and vertical bars represent the interquartile range across 250 simulations. Dashed line and shaded region show median estimate and interquartile range for the model scenario without risk heterogeneity.

Figure S15. Reductions from 2015 to 2030 in (A) incidence of all infections (including superinfections), (B) incidence of new infections, and (C) prevalence of active infection by relative rate of treatment in the high-risk class compared to low- and moderate-risk classes. Points represent median estimate and vertical bars represent the interquartile range across 250 simulations. Dashed line and shaded region show the median and interquartile range of reductions for the model without risk heterogeneity.

Figure S16. Total treatment courses delivered versus new infections averted from 2015 to 2030 for each model simulation, by the relative rate (RR) of treatment in the high-risk class compared to low- and moderate-risk classes. Black points represent the model without risk heterogeneity. The clusters reflect simulations from various treatment scenarios (treatment rate 10, 30, or 60 courses per 100 PY).

Figure S17. Prevalence of active infection overall and in each risk class by the relative rate (RR) of treatment in the high-risk class compared to low- and moderate-risk classes. Lines represent median estimate and shaded regions represent the interquartile range across 250 simulations.

Figure S18. (A) All infections (including superinfections) and (B) new infections averted from 2015 to 2030 per 1,000 treatment courses delivered by the relative rate (RR) of treatment in the high-risk class compared to low- and moderate-risk classes in a scenario with risk class-assortative mixing (within class contacts 25% more likely than between class contacts). Points represent median estimate and vertical bars represent the interquartile range across 250 simulations. Dashed line and shaded region represent the median and interquartile range of reductions for the model with random mixing between classes.

Figure S19. Reductions from 2015 to 2030 in (A) incidence of all infections (including superinfections), (B) incidence of new infections, and (C) prevalence of active infection by relative rate of treatment in the high-risk class compared to low- and moderate-risk classes in a scenario with risk class-assortative mixing (within class contacts 25% more likely than between class contacts). Points represent median estimate and vertical bars represent the interquartile range across 250 simulations. Dashed line and shaded region show the median and interquartile range of reductions for the model with random mixing between classes..

Figure S20. Prevalence of active infection overall and in each risk class by the relative rate (RR) of treatment in the high-risk class compared to low- and moderate-risk classes with a greater proportion of population (33%) in the high-risk class. Lines represent median estimate and shaded regions represent the interquartile range across 250 simulations.

Figure S21. (A) All infections (including superinfections) and (B) new infections averted from 2015 to 2030 per 1,000 treatment courses delivered by the relative rate (RR) of treatment in the high-risk class compared to low- and moderate-risk classes in a scenario with a greater proportion of population (33%) in the high-risk class. Points represent median estimate and vertical bars represent the interquartile range across 250 simulations. Dashed line and shaded region represent the median and interquartile range of reductions for the model with original population distribution (15% high-risk).

Figure S22. Reductions from 2015 to 2030 in (A) incidence of all infections (including superinfections), (B) incidence of new infections, and (C) prevalence of active infection by relative rate of treatment in the high-risk class compared to low- and moderate-risk classes in a scenario with a greater proportion of population (33%) in the high-risk class. Points represent median estimate and vertical bars represent the interquartile range across 250 simulations. Dashed line and shaded region show the median and interquartile range of reductions for the model with original population distribution (15%).

Figure S23. Reductions from 2015 to 2030 in (A) incidence of all infections (including superinfections), (B) incidence of new infections, and (C) prevalence of active infection by relative rate of treatment in the high-risk class compared to low- and moderate-risk classes by the post-treatment reduction in HCV risk. Points represent median estimate and vertical bars represent the interquartile range across 250 simulations. Dashed line and shaded region show the median and interquartile range of reductions for the model without risk heterogeneity.

**

Figure S24. Annual incidence of reinfection per 100 person-years overall and in each risk class by post-treatment reduction in HCV risk. Lines represent median estimate and shaded regions represent the interquartile range across 250 simulations.

Figure S25. All infections (including superinfections) averted from 2015 to 2030 per 1,000 treatment courses delivered in scenarios in scenarios with treatment pulses of either 60 or 80 courses per 100 PY for 6 months, 1 year, or 2 years, compared to a baseline scenario of constant treatment of 10 per 100 PY. Points represent median estimate and vertical bars represent the interquartile range across 250 simulations.

Figure S26. Reductions from 2015 to 2030 in (A) incidence of all infections (including superinfections), (B) incidence of new infections and (C) prevalence of active infection in scenarios in scenarios with treatment pulses of either 60 or 80 courses per 100 PY for 6 months, 1 year, or 2 years, compared to a baseline scenario of constant treatment of 10 per 100 PY. Points represent median estimate and vertical bars represent the interquartile range across 250 simulations.

Figure S27. Total treatment courses delivered versus new infections averted from 2015 to 2030 for each model simulation of scenarios with treatment pulses of either 60 or 80 courses per 100 PY for 6 months, 1 year, or 2 years. The clusters represent simulations from various treatment scenarios, including constant treatment (i.e., no pulse; black dots) of 10, 30, 60, and 90 courses per 100 PY.

**REFERENCES**

1. Genberg, B. L. *et al.* Trajectories of injection drug use over 20 years (1988-2008) in Baltimore, Maryland. *Am. J. Epidemiol.* **173**, 829–836 (2011).

2. Sun, J. *et al.* Mortality among people who inject drugs: a prospective cohort followed over three decades in Baltimore, MD, USA. *Addiction* **117**, 646–655 (2022).

3. Martin, N. K., Hickman, M., Hutchinson, S. J., Goldberg, D. J. & Vickerman, P. Combination interventions to prevent HCV transmission among people who inject drugs: modeling the impact of antiviral treatment, needle and syringe programs, and opiate substitution therapy. *Clin. Infect. Dis. Off. Publ. Infect. Dis. Soc. Am.* **57 Suppl 2**, S39-45 (2013).

4. Aisyah, D. N., Shallcross, L., Hully, A. J., O’Brien, A. & Hayward, A. Assessing hepatitis C spontaneous clearance and understanding associated factors—A systematic review and meta-analysis. *J. Viral Hepat.* **25**, 680–698 (2018).

5. Grebely, J. *et al.* Hepatitis C virus clearance, reinfection, and persistence, with insights from studies of injecting drug users: towards a vaccine. *Lancet Infect. Dis.* **12**, 408–414 (2012).

6. Micallef, J. M., Kaldor, J. M. & Dore, G. J. Spontaneous viral clearance following acute hepatitis C infection: A systematic review of longitudinal studies. *J. Viral Hepat.* **13**, 34–41 (2006).

7. Mehta, S. H. *et al.* Protection against persistence of hepatitis C. *The Lancet* **359**, 1478–1483 (2002).

8. Osburn, W. O. *et al.* Spontaneous Control of Primary Hepatitis C Virus Infection and Immunity Against Persistent Reinfection. *Gastroenterology* **138**, 315–324 (2010).

9. Mondelli, M. U., Cerino, A. & Cividini, A. Acute hepatitis C: diagnosis and management. *J. Hepatol.* **42**, S108–S114 (2005).

10. Hofer, H. *et al.* Spontaneous viral clearance in patients with acute hepatitis C can be predicted by repeated measurements of serum viral load. *Hepatology* **37**, 60–64 (2003).

11. Nephew, L. D. *et al.* Removal of medicaid restrictions were associated with increased hepatitis C virus treatment rates, but disparities persist. *J. Viral Hepat.* **29**, 366–374 (2022).

12. Iversen, J. *et al.* Estimating the cascade of hepatitis C testing, care and treatment among people who inject drugs in Australia. *Int. J. Drug Policy* **47**, 77–85 (2017).

13. Mirzazadeh, A. *et al.* Progress toward closing gaps in the hepatitis C virus cascade of care for people who inject drugs in San Francisco. *PLOS ONE* **16**, e0249585 (2021).

14. Falade-Nwulia, O. *et al.* Barriers and facilitators of hepatitis C treatment uptake among people who inject drugs enrolled in opioid treatment programs in Baltimore. *J. Subst. Abuse Treat.* **100**, 45–51 (2019).

15. Hajarizadeh, B. *et al.* Direct-acting antiviral treatment for hepatitis C among people who use or inject drugs: a systematic review and meta-analysis. *Lancet Gastroenterol. Hepatol.* **3**, 754–767 (2018).

16. Graf, C. *et al.* Efficacy of Direct-acting Antivirals for Chronic Hepatitis C Virus Infection in People Who Inject Drugs or Receive Opioid Substitution Therapy: A Systematic Review and Meta-analysis. *Clin. Infect. Dis.* **70**, 2355–2365 (2020).

17. Latham, N. H. *et al.* Staying hepatitis C negative: A systematic review and meta‐analysis of cure and reinfection in people who inject drugs. *Liver Int.* **39**, 2244–2260 (2019).

18. Caven, M., Malaguti, A., Robinson, E., Fletcher, E. & Dillon, J. F. Impact of Hepatitis C treatment on behavioural change in relation to drug use in people who inject drugs: A systematic review. *Int. J. Drug Policy* **72**, 169–176 (2019).

19. Bruneau, J. *et al.* Sustained drug use changes after hepatitis c screening and counseling among recently infected persons who inject drugs: A longitudinal study. *Clin. Infect. Dis.* **58**, 755–761 (2014).

20. Aspinall, E. J. *et al.* Does informing people who inject drugs of their hepatitis C status influence their injecting behaviour? Analysis of the Networks II study. *Int. J. Drug Policy* **25**, 179–182 (2014).
